# WaSH insecurity and anxiety among people who inject drugs in the Tijuana-San Diego border region

**DOI:** 10.1186/s12889-023-17341-9

**Published:** 2024-01-02

**Authors:** Lourdes Johanna Avelar Portillo, Alhelí Calderón-Villarreal, Daniela Abramovitz, Alicia Harvey-Vera, Susan Cassels, Carlos F. Vera, Sheryl Munoz, Arturo Tornez, Gudelia Rangel, Steffanie A. Strathdee, Georgia L. Kayser

**Affiliations:** 1 Herbert Wertheim School of Public Health and Human Longevity Science, University of California San Diego, La Jolla, CA USA; 2 Benioff Homelessness and Housing Initiative, School of Medicine, University of California San Francisco, San Francisco, CA USA; 3 Department of Family and Preventive Medicine, University of California, San Diego, La Jolla, CA USA; 4 School of Public Health, San Diego State University, San Diego, CA USA; 5 Department of Medicine, Division of Infectious Diseases and Global Public Health, University of California San Diego, La Jolla, CA USA; 6 Facultad de Medicina, Campus Tijuana, Universidad de Xochicalco, Tijuana, Baja California México; 7 United States-Mexico Border Health Commission, Tijuana, Baja California Mexico; 8 Department of Geography, University of California Santa Barbara, Santa Barbara, CA USA; 9 Departamento de Estudios de Población, Colegio de La Frontera Norte, Tijuana, México

**Keywords:** Water, sanitation, and hygiene (WaSH), WaSH insecurity, Homelessness, PWID, Psychosocial distress, Anxiety, Health inequities

## Abstract

**Background:**

Water, sanitation, and hygiene (WaSH) insecurity increases the risk of water-related diseases. However, limited research has been conducted on psychosocial distress as it relates to WaSH insecurity, especially among people who inject drugs (PWID). We examined the relationship between WaSH insecurity and related anxiety among PWID living in different housing conditions along the US-Mexico border region.

**Methods:**

From 2020–2021, a cross-sectional study was conducted among 585 people who injected drugs within the last month in Tijuana (*N* = 202), San Diego (*N* = 182), and in both Tijuana and San Diego (*N* = 201). Participants underwent interviewer-administered surveys related to WaSH access, substance use, and generalized anxiety disorder (GAD-7). Quasi-Poisson regressions were used to assess associations between WaSH insecurity and anxiety in the prior 6-months.

**Results:**

Participants were 75% male, 42% were unhoused and 91% experienced WaSH insecurity in the prior 6-months. After adjusting for housing status, gender, and age, lack of access to basic drinking water (Adj RR: 1.28; 95% CI: 1.02–1.58), sanitation (Adj RR:1.28; 95% CI: 1.07–1.55), and a daily bath/shower (Adj RR: 1.38; 95% CI: 1.15–1.66) were associated with mild-severe anxiety. The number of WaSH insecurities was independently associated with a 20% increased risk of experiencing anxiety per every additional insecurity experienced (Adj RR: 1.20; CI: 1.12–1.27). We also found a significant interaction between gender and housing status (*p* = 0.003), indicating that among people experiencing sheltered/unsheltered homelessness, women had a higher risk of mild-severe anxiety compared to men (Adj RR: 1.55; 95% CI: 1.27–1.89). At the same time, among women, those who are unhoused have 37% increased risk of anxiety than those who live in stable housing conditions (Adj RR: 1.37; 95% CI: 1.01–1.89).

**Conclusion:**

The lack of specific WaSH services, particularly lack of drinking water, toilets, and daily showers were associated with higher levels of anxiety among PWID in the Tijuana-San Diego border region. Women experiencing homelessness were especially vulnerable. WaSH interventions that provide safe, 24-h access may help to reduce anxiety and health risks associated with WaSH insecurity.

## Introduction

Access to basic water, sanitation, and hygiene (WaSH) is critical for reducing the spread of enteric and infectious diseases caused by bacteria, viruses, helminths, and protozoa. Studies have shown that WaSH insecurity (or a lack of basic water, sanitation and hygiene services) can increase the prevalence of diarrhoea [1–3], chronic dehydration [4, 5], urinary tract infections [6–8], skin infections [9, 10], and increase the risk of respiratory diseases associated with limited access to clean water and hygiene [11–13]. While the impacts of WaSH insecurity on physical health have been established in the public health realm, there is limited research on the mental health impacts of WaSH insecurity, especially among people who inject drugs (PWID). WaSH insecurity can produce profound psychosocial distress, (e.g., stress, anxiety and depression) further impacting the quality of life for people who already live in marginalized conditions [14–18].

In most WaSH studies related to mental health, psychosocial distress has been observed during water shortages or with drinking water contamination, at the household level, and intersect with gender and class disparities. In the ethnographic work of Farhana Sultana (2011), for example, psychosocial distress is reflected through the notion of “suffering from water” invoked by men and women who used water from wells contaminated with arsenic in Bangladesh [16]. Sultana argues that distress is produced and shaped through the everyday survival struggles, and navigating access or lack thereof, use, and control over water resources (p. 164). In Bolivia, a study found that limited access to drinking water services at the household level resulted in higher levels of psychosocial distress outcomes among women [19]. The intersection of gender, WaSH insecurity, and psychosocial distress is also seen in the work of Stevenson et al.’s (2016) study in Ethiopia; they found that water insecurity is an important predictor of psychosocial distress and access to water can help improve women’s mental well-being [20]. In Haiti, a study also concluded that households with poor water quality and quantity had higher levels of depression and anxiety, and that men in particular exhibited higher levels of anxiety and depression compared to women [21]. In other studies, lack of sanitation services, specifically open defecation coping practices were associated with psychosocial distress, suggesting that the lack of sanitation services increases the risk of psychosocial distress [22–24]. Thus, psychosocial distress manifests in everyday roles and experiences and is conceptualized as an outcome that often arises from limited access to WaSH services.

Currently, existing literature and even global water monitoring efforts focus on the household level [25]. While limited, there are a few studies that move beyond the household level and explore the impacts of WaSH insecurity among unhoused communities [26–31]. The work of Calderón-Villarreal et al. (2022), for instance, examines the lived experiences of the Tijuana River communities, many of whom are PWID and unhoused. However, no study has addressed the intersections of WaSH insecurity and anxiety among PWID, many of whom are unhoused or live in unstable housing conditions. Psychosocial distress among PWID is common given the stigmatized nature of injection drug use; PWID are often disconnected from services and social networks, and experience police harassment [32–34].

Examining the association between WASH insecurity and anxiety among PWID in the Tijuana-San Diego border region is important given the contrasting vulnerabilities and socioeconomic differences that exist between these two regions despite their proximity. The border communities of the Tijuana-San Diego metropolitan area are highly vulnerable to infectious diseases due to large population movement for work, school, medical care, leisure, and substance use [35–42]. The border region is also an area with high levels of injection drug use and with ever changing policy/law enforcement responses to substance use [41, 43]. Many PWID living in these communities are migrants deported from the United States through Mexican border cities, such as Tijuana who experience homelessness and as a result may be in higher risk for WaSH insecurity and poorer health outcomes [28, 42, 44]. To date, relatively little is known about the WaSH insecurity experiences among PWID living in different housing conditions in the border region, apart from Calderón-Villarreal et al. (2022). Given that WaSH insecurity is closely embedded in prolonged homelessness and poverty, health disparities, and environmental injustice issues [5, 28, 45, 46] examining the impact of WaSH insecurity and psychosocial outcomes among unhoused PWID in these two communities is important as part of harm reduction efforts to improve quality of health and well-being.

Our study provides new insight into understanding the WaSH insecurity experiences of PWID living in varying housing conditions in the Tijuana-San Diego region. We hypothesized that anxiety is significantly associated with poor WaSH access. Second, we hypothesized that differences exist among the sample population and unhoused women who inject drugs are more likely to have a higher risk of anxiety compared to unhoused males. This is based on existing literature at the household level that highlights the burden women bear collecting water and psychosocial distress resulting from inadequate access to WaSH services [16, 20, 47]. Lastly, we hypothesized that people residing in Tijuana are more likely to report higher levels of anxiety related to WaSH insecurity than those people residing in San Diego. Ultimately, this study seeks to advance our understanding of the psychosocial distress, specifically the anxiety outcome, related to WaSH insecurity for PWID on a binational level.

## Methods

### Study design and population

This is a cross-sectional study and part of the parent cohort study, “La Frontera” (R01DA049644; PI: Strathdee), conducted in San Diego County, United States of America and Tijuana, Baja California, Mexico between October 2020 to September 2021. Inclusion criteria included people aged ≥ 18 who reported injection drug use within the prior month and live in San Diego County or Tijuana. [48]. Participants were also eligible if they resided in San Diego and reported having crossed the border to inject drugs in Tijuana within the last two years. Recruitment took place in parks, shelters, streets, canals, and vacant lots, as previously described [48, 49]. Interviewer-administered surveys were conducted in either Spanish or English. Participants were administered two surveys, one at the baseline visit containing different demographic and injection drug use information. The second was a supplemental survey visit, containing WaSH insecurity and anxiety measurements, which was administered approximately one week after the baseline visit to reduce participant burden. Only participants that provided written informed consent, completed the baseline and supplemental surveys visits, and responded to questions related to anxiety and WaSH comprised the analytic sample for this study.

### Ethics statement

The study activities were reviewed and approved by the Office of Institutional Review Board (IRB) at the University of California San Diego and Xochicalco University (IRB # 191390). All study activities were carried out in accordance with IRB guidelines and regulations.

### Measures

Survey measures collected at the baseline and supplemental interview visits included sociodemographic factors, housing status and other individual vulnerabilities such as physical violence experienced in the prior 6-months. In this study, housing status was measured based on three categories: permanently housed, sheltered homelessness, and unsheltered homelessness. Participants who reported living in emergency shelters, temporary shelters, rented motel rooms, and refugee camps, were classified as experiencing sheltered homelessness. Participants who reported living on the street, in abandoned buildings, shooting galleries, and vehicles, were classified as living in unsheltered conditions (unsheltered homelessness). In the regression models, those experiencing sheltered/unsheltered homelessness were grouped as people living in unstable housing conditions and the reason being that we are assuming people in these two categories may not have continuous and reliable access to WaSH services. Anyone who reported living in their own apartment/home, with family members, or friends were categorized as living in permanent or stable housing conditions.

Generalized anxiety disorder (GAD) was measured using the GAD-7 scale, which is a 7-item instrument that assesses the severity of anxiety symptoms over a two-week period. This scale has been validated in previous studies [50, 51] and has demonstrated validity in the same cohort population [49].

To assess forms of WaSH insecurity, we use validated instruments adapted from the Joint Monitoring Programme (JMP) definitions and standardized classification of WaSH facilities [52]. The JMP uses a standard facility type classification to compare progress between countries. In this study, we measure a person’s ability to have basic access to WaSH services in the past six months. First, basic drinking water access was defined as whether a person had access to improved water sources within a 30-min or less roundtrip distance to their sleeping location. Basic sanitation access considered whether a person had access to improved sanitation facilities that are not shared with other households. If a person indicated using an outdoor setting, including open defecation, this was categorized as a lack of access to basic sanitation. Lastly, basic hygiene access was considered according to two categories: hand-hygiene and body-hygiene practices. Basic hand-hygiene was based on whether a person had access to soap and water in their living environment (moving beyond the household level to make advancements in global JMP reports) [53]. While the JMP does not capture body-hygiene accessibility, we included this measurement by considering daily access to a shower or bath. The inclusion of daily access to a shower or bath in the analysis is important because existing literature has shown that limited access to a bath or shower can impact a person’s self-esteem, appearance, and increases experiences of stigma and exclusion, and is especially important for the unhoused [7, 31, 54]. Lastly, since our study population were PWID, we included the measurement of basic access to improved water sources used for drug preparation and cleaning wounds or abscesses to highlight the need for services that can help reduce the risk of infections. In this study, WaSH insecure participants were defined as those with limited WaSH access, access to unimproved water sources for drinking and drug preparation, unimproved sanitation sources, or were forced to embark in risky coping strategies (e.g., open defecation, drinking water from a canal, river, streams, and lack of daily shower/bath, etc.). If participants reported a lack of basic access to any of these WaSH services, then they were classified as experiencing WaSH insecurity, which we used to create a variable representing the number of WaSH insecurities ranging from zero to six (maximum number of services examined).

### Data analysis

The main outcome for this study is whether a person experienced generalized anxiety (mild-severe vs. none-minimal) according to the GAD-7 score, where values of five or greater represented mild to severe levels of anxiety and values smaller than 5 represented minimal to no anxiety [51]. The reasoning for choosing this benchmark to measure GAD-7 with a cut-off point of five is because it appeared to be optimal for detecting GAD in this particular sample population, and other studies have used similar benchmarks when measuring GAD in housed adults [55–57].

Chi-square tests for independence were performed to assess the relationship between WaSH insecurity, socio-demographic characteristics of participants (including housing status, gender identity, city of residence, and age group to name a few), and the outcome of anxiety to determine associations. Univariate and multivariable generalized linear models, leveraging a quasi-Poisson distribution with robust variance estimation and a log link, were used to explore potential risk factors for the dichotomous outcome (presence or absence of mild-severe anxiety). We use quasi-Poisson because it has shown to perform better then log-binomial at estimating Risk Ratios and account for over-dispersion in data [58]. The multivariable quasi-Poisson regression models were used to identify the correlates of anxiety and to investigate if anxiety is associated with WaSH insecurity experiences, after controlling for socio-demographic characteristics.

All variables in the univariate regression models listed in Table 3 were considered for inclusion in the multivariable models. The final multivariable model was constructed by taking into account subject matter relevance, existing literature, statistical significance at the univariate level, and relationships among potential predictors (e.g., correlations, confounding, and interactions). The final model included the WaSH variables of interest that were not highly correlated with each other plus covariates that retained a 0.10 significance level. In the multivariable model, we also tested for interaction items, including the interaction between housing status and gender given that one of our hypotheses was whether housing moderated the relationship between gender and anxiety. No multi-collinearity problems existed in our final models, as indicated by the variance inflation factor scores that were below five [59]. All statistical analyses were conducted using R version 4.2.2 and RStudio Desktop 2022.12.0 + 353.

## Results

### Population characteristics

Of 612 study participants, 585 completed both baseline and supplemental interviews and responded to questions about GAD-7 and WaSH access, and thus comprised the analytic sample. Among the 585 participants, all injected within the past month in Tijuana only (*N* = 202), San Diego only (*N* = 192), or both cities (*N* = 201). Respondents were predominantly male (75%) with an average age of 43 years (SD ± 11). Roughly forty-three percent reported living in unsheltered conditions. Approximately 73% of participants were Latinx/Hispanic, of which 33% resided in Tijuana and 40% in San Diego. Of those participants who reported living in Tijuana, twenty-seven percent reported being deportees from the United States. In the sample, nine percent reported sex work in the prior six months.

Table 1 reports the GAD-7 results among the analytic sample on a range (None-Minimal, Mild, Moderate, or Severe) and the dichotomous outcome (None-Minimal or Mild-Severe) used in the analytical models. When assessing anxiety as dichotomous on the GAD-7 Scale, 55% of participants reported experiencing anxiety in the range between mild and severe (5-21), while roughly 45% reported having no or minimal anxiety (0–4). One participant that failed to provide a complete response was excluded from the analytic sample and the final analysis. On average, the study participants reported a score of 6.2 (± 6) on the GAD-7 scale.

**Table 1:** Generalized anxiety disorder outcome in the analytic sample (*N* = 585)

Characteristic	Count (%)
Anxiety scale	
0 – 4: None-Minimal	261 (44.5)
5 – 9: Mild	183 (31.2)
10 – 14: Moderate	80 (13.7)
15 – 21: Severe	61 (10.4)
Missing	1 (0.2)
Dichotomous anxiety scale^a^	
0 – 4: None-Minimal	261 (44.6)
5 – 21: Mild-Severe	324 (55.4)

^a^Missing value was not included in final sample population and study analysis. The dichotomous anxiety scale shown is the outcome variable used in regression models, excluding the missing value

Table 2 shows demographic characteristics by anxiety outcome. Compared to participants who experienced none to minimal anxiety, those with mild-severe anxiety were more likely to be younger, unhoused, and experiencing WaSH insecurity. In the sample, among those who reported mild-severe anxiety 72% were male, whereas 78% among those who reported none-minimal anxiety were male. Among those experiencing anxiety, 62% resided in San Diego and 38% percent of participants resided in Tijuana. Furthermore, 47% among those who reported mild-severe anxiety were unhoused as compared to 37% unhoused among those with none-minimal anxiety (*p* < 0.05).

**Table 2:** Population demographic characteristics by anxiety outcome (*N* = 585)

Characteristics	Anxiety *N* = 585		*P*-value^b^
	None-Minimal^a^ *n* = 261 (100%)	Mild-Severe^a^ *n* = 324 (100%)	
Gender identity			**0.10**
Male	203 (77.78)	233 (71.91)	
Female^c^	58 (22.22)	91 (28.09)	
Age			**0.04**
Younger than 36	57 (21.84)	92 (28.40)	
36 to 43	68 (26.05)	91 (28.09)	
44 to 52	61 (23.37)	79 (24.38)	
Older than 52	75 (28.74)	62 (19.14)	
City			**0.07**
San Diego	181 (69.35)	202 (62.35)	
Tijuana	80 (30.65)	122 (37.65)	
Housing Status			**0.04**
Permanent	100 (38.31)	112 (34.57)	
Sheltered homelessness	64 (24.52)	60 (18.52)	
Unsheltered homelessness	97 (37.16)	152 (46.91)	
Latinx/Hispanic/Mexican			**0.79**
Yes	190 (72.80)	239 (73.77)	
No	71 (27.20)	85 (26.23)	
Engaged in sex work, past 6 months			**0.005**
Yes	14 (5.38)	39 (12.04)	
No	246 (94.62)	285 (87.96)	
Physical violence past 6 months			**0.002**
Yes	37 (14.18)	79 (24.38)	
No	224 (85.82)	245 (75.62)	
Law enforcement violence past 6 months			0.225
Yes	24 (9.20)	40 (12.35)	
No	237 (90.80)	284 (87.65)	
Stopped (arrested/let go) by police past 6 months			**0.03**
Yes	70 (26.82)	113 (34.88)	
No	191 (73.18)	211 (65.12)	
WaSH insecurity^d^			
Drinking water insecurity	19 (7.28)	44 (13.62)	**0.01**
Sanitation insecurity	168 (64.86)	249 (76.85)	** < 0.001**
Bathing insecurity	150 (57.47)	241 (74.38)	** < 0.001**
Hand hygiene insecurity	144 (55.17)	221 (68.21)	** < 0.001**
Water insecurity for drug preparation	14 (5.36)	39 (12.04)	**0.005**
Water insecurity for cleaning wounds/abscesses	19 (7.31)	32 (9.91)	0.27
Number of WaSH insecurities			
Median (IQR)	2 (1 – 3)	3 (2 – 3)	** < 0.001**^e^

^a^Individual demographic characteristics were summarized across column total percentages per category

^b^*P* values were generated using χ2 tests

^c^Only one participant was trans female

^d^WaSH insecurity proportions only reflect those participants who reported yes to experiencing different forms of WaSH insecurity

^e^The *p*-value for the number of WaSH insecurities was calculated using a Mann–Whitney U test

WaSH insecurity in this study was defined as not having basic access to improved water sources for drinking water, hand hygiene, water for cleaning wounds, and water for drug preparation in the past six months. At the same time, our WaSH insecurity measurements included not having access to improved sanitation facilities that are not shared with other households and the inability to shower or bathe daily. Eighty-one percent of respondents reported at least three forms of WaSH insecurity, and only eight percent reported experiencing no WaSH insecurity in the past six months. The most common forms of WaSH insecurity reported were sanitation (72%) and bathing (67%). Participants in the mild-severe anxiety group were more likely to report a lack of basic sanitation as compared to participants in the none-minimal anxiety group (roughly 77% vs. 65% respectively; *p* < 0.001). Similarly, we found that 74% of the participants who reported mild-severe anxiety were likely lacking access to daily shower or bathing facilities as compared to 57% of the participants with none-minimal anxiety (*p* < 0.001).

### WaSH insecurity and generalized anxiety disorder

In the final models, the independent variables included different WaSH services that may be associated with the risk of anxiety. Distinguishing among each service was important to highlight specific services needed in the community and implications for psychosocial outcomes, in this case anxiety, that may be impacting the mental well-being of the study population. Furthermore, the final models controlled for different demographic variables, since we were interested in examining whether the risk of anxiety is greater or lower depending on their gender identity, age group, and people’s housing status. All of these variables remain heavily underexplored in existing literature. Results from the univariate models assessing whether there is an association between WaSH insecurity, demographic factors, and anxiety are presented in Table 3. The risk of experiencing anxiety for people who did not have basic access to drinking water was 30% higher compared to those that had basic drinking water access (RR: 1.30; 95% CI: 1.05–1.61). People who lacked access to basic sanitation had higher risk of experiencing mild-severe anxiety compared to those participants that had access to basic sanitation (RR: 1.32; 95% CI: 1.12–1.57). Furthermore, people who did not have access to a daily bath/shower had 44% higher risk of experiencing anxiety than people who had daily access to a bath/shower (RR: 1.44; 95% CI: 1.22–1.71). We found community differences with people residing in Tijuana reporting 15% increased risk of anxiety compared to those residing in San Diego (RR: 1.15; CI: 0.98–1.33). Lastly, those who reported experiencing recent physical violence (in past six months) had 30% higher risk of anxiety compared to those who did not experience any recent physical violence (RR: 1.30; CI: 1.10–1.54) at α = 0.10.

**Table 3:** Characteristics associated with mild-severe anxiety among PWID on the Tijuana-San Diego border (univariate models)

Baseline variable	Category	RR	95% CI	*P*-value
WaSH insecurity	Drinking water insecurity (yes vs. no)	1.30	1.05 – 1.61	**0.014**
	Sanitation insecurity (yes vs. no)	1.32	1.12 – 1.57	**0.002**
	Bathing insecurity (yes vs. no)	1.44	1.22 – 1.71	** < 0.001**
	Hand hygiene insecurity (yes vs. no)	1.29	1.11 – 1.51	**0.001**
	Water insecurity for preparing drugs (yes vs. no)	1.37	1.09 – 1.71	**0.005**
	Water insecurity for cleaning wounds/abscesses (yes vs. no)	1.15	0.89 – 1.45	0.271
	Number of WaSH insecurities	1.18	1.11 – 1.24	** < 0.001**
Housing	Unstably housed (yes vs. no)	1.08	0.92 – 1.26	0.350
Gender	Female (yes vs. no)	1.14	0.97 – 1.34	**0.106**
City	Tijuana (yes vs. no)	1.15	0.98 – 1.33	**0.077**
Age groups	18–35 (Age 52 + : ref group)	1.36	1.10 – 1.70	**0.005**
	36–43 (Age 52 + : ref group)	1.26	1.02 – 1.57	**0.033**
	44–52 (Age 52 + : ref group)	1.25	1.00 – 1.56	**0.052**
Engaged in sex work in the past 6 months	Sex work (yes vs. no)	1.37	1.09 – 1.70	**0.006**
Race/ethnicity	Black/African American (White: ref group)	0.88	0.57 – 1.31	0.533
	Latinx/Hispanic (White: ref group)	1.02	0.84 – 1.24	0.869
	Other^a^ (White: ref group)	1.08	0.74 – 1.55	0.680
Physical violence in the prior 6-months	Experienced violence by anyone (yes vs. no)	1.30	1.10 – 1.54	**0.002**
	Experienced violence by law enforcement (yes vs. no)	1.15	0.91 – 1.42	0.226
Law enforcement interaction in the prior 6-months	Stopped (arrested/let go) by police (yes vs. no)	1.18	1.01 – 1.37	**0.037**

Basic access to drinking water, sanitation, and hand hygiene are adapted definitions from the WHO/UNICEF Joint Monitoring Programme. The notation RR = crude risk ratios; CI = confidence interval. Significance denoted at α = 0.10

^a^Denotes combined race groups for those groups with small sample size

Table 4 summarizes the multivariable model results examining the relationship between WaSH insecurity and anxiety. In the final model, the independent variables included WaSH variables of interest (drinking water, sanitation, bathing, and handwashing services) that were not highly correlated with each other. The multivariable model results on Table 4 suggest that the lack of access to basic drinking water (Adj RR: 1.28; 95% CI: 1.02–1.58), sanitation (Adj RR:1.28; 95% CI: 1.07–1.55), and bathing/showering facilities (Adj RR: 1.38; 95% CI: 1.15–1.66), were independently associated with mild-severe anxiety after adjusting for housing status, gender, and age groups. We also found a marginal significant difference between people who reported being victims of physical violence in the past six months at *p* = 0.051, indicating that those who have experienced recent physical violence are 19% more likely to experience anxiety compared to those who have not experienced any recent physical violence. Participants who were thirty-five and younger were 30% more likely to experience anxiety (Adj RR:1.30; 95% CI: 1.05–1.62), compared to people older than 52 years. There was a statistically significant interaction between housing and gender (*p*-value = 0.005) shown in Table 4. After evaluating the main effects of the variables involved in the interaction on the outcome, among participants who reported being unstably housed (experiencing homelessness), females had 1.5 times the risk of experiencing mild-severe anxiety compared to unhoused male participants (RR: 1.55; 95% CI:1.26–1.89).

**Table 4:** WaSH insecurity and population demographic correlates of mild-severe anxiety among PWID on the Tijuana-San Diego border (multivariable model)

Baseline variable	Category	Adj. RR	95% CI	*P*-value
WaSH insecurity	Drinking water insecurity (yes vs. no)	1.28	1.02 – 1.58	**0.027**
	Sanitation insecurity (yes vs. no)	1.28	1.07 – 1.55	**0.009**
	Bathing insecurity (yes vs. no)	1.38	1.15– 1.66	**0.001**
	Hand hygiene insecurity (yes vs. no)	1.13	0.96 – 1.33	0.157
Housing^a^	Unstably housed (yes vs. no)	0.79	0.66 – 0.95	**0.014**
Gender^a^	Female (yes vs. no)	0.92	0.67 – 1.24	0.601
Age groups	18–35 (Age 52 + : ref group)	1.30	1.05 – 1.62	**0.017**
	36–43 (Age 52 + : ref group)	1.20	0.97 – 1.49	0.102
	44–52 (Age 52 + : ref group)	1.25	1.00 – 1.57	**0.049**
Physical violence in the prior 6-months	Experienced violence by anyone (yes vs. no)	1.19	1.00 – 1.57	**0.051**
Interaction item	Unstably housed*female	1.68	1.18 – 2.42	**0.005**
	Among females: Unstably housed (yes vs. no)	1.33	0.97 – 1.84	**0.079**
	Among males: Unstably housed (yes vs. no)	0.79	0.66 – 0.95	**0.014**
	Among unstably housed: Female vs. Male	1.55	1.26 – 1.89	** < 0.001**
	Among NOT unstably housed: Female vs. Male	0.92	0.67 – 1.24	0.596

The notation Adj. *RR* adjusted risk ratios, *CI* confidence interval. Significance denoted at α = 0.10

The interaction item in this multivariable model is denoted as unstably housed*female

^a^The corresponding housing and gender RR estimates cannot be interpreted directly because of their significant interaction

Lastly, we included in a multivariable model a variable that sums the number of WaSH insecurities participants reported experiencing (ranging from zero to six, the total number of services examined). Table 5 highlights these results where we found that for every additional increase in WaSH insecurities a person experiences, the risk of anxiety increases by 20% (Adj RR: 1.20; CI: 1.121.27). The same variables that were significant in our model shown in Table 4 remained significant in this new model. We still found that younger adults and people who experienced recent physical violence had higher risk of anxiety. Lastly, the interaction between gender and housing remained statistically significant (*p* = 0.003). After evaluating the main effects of the variables involved in the interaction on the outcome, among participants who experiencing homelessness, females had 1.5 times the risk of experiencing mild-severe anxiety compared to unhoused male participants (RR: 1.55; 95% CI:1.27–1.89). Among female participants, those experiencing homelessness (or unstably housed) had 37% higher risk of anxiety (RR: 1.37; 95% CI: 1.01–1.89) than females living in permanent housing conditions. Furthermore, counter to expectations, among male participants, those who were unstably housed had 21% lower risk of experiencing anxiety compared to males living in stable housing conditions (RR: 0.79; 95% CI: 0.66–0.96). In other words, men in stable housing conditions had higher risk of experiencing anxiety compared to unstably housed men.

**Table 5:** Number of WaSH insecurities and correlates of mild-severe anxiety among PWID on the Tijuana-San Diego border (multivariable model)

Baseline variable	Category	Adj. RR	95% CI	*P*-value
WaSH insecurity	Number of WaSH insecurities	1.20	1.12 – 1.27	** < 0.001**
Housing^a^	Unstably housed (yes vs. no)	0.79	0.66 – 0.96	**0.015**
Gender^a^	Female (yes vs. no)	0.90	0.66 – 1.20	0.485
Age groups	18–35 (Age 52 + : ref group)	1.30	1.04 – 1.61	**0.020**
Age groups	36–43 (Age 52 + : ref group)	1.21	0.98 – 1.51	0.084
	44–52 (Age 52 + : ref group)	1.24	0.99 – 1.55	0.060
Physical violence in the prior 6-months	Experienced violence by anyone (yes vs. no)	1.19	1.00 – 1.41	**0.050**
Interaction item	Unstably housed*female	1.73	1.21 – 2.49	**0.003**
	Among females: Unstably housed (yes vs. no)	1.37	1.01 – 1.89	**0.048**
	Among males: Unstably housed (yes vs. no)	0.79	0.66 – 0.96	**0.015**
	Among unstably housed: Female vs. Male	1.55	1.27 – 1.89	** < 0.001**
	Among NOT unstably housed: Female vs. Male	0.90	0.66 – 1.20	0.485

The notation Adj. *RR* adjusted risk ratios, *CI* confidence interval. Significance denoted at α = 0.05

The interaction item in this multivariable model is denoted as unstably housed*female

^a^The corresponding housing and gender RR estimates cannot be interpreted directly because of their significant interaction

## Discussion

This is the first study to explore the association between WaSH insecurity and anxiety among PWID in the Tijuana-San Diego metropolitan region. In this study, we found there was a high prevalence of anxiety among our study sample, with more than half reporting mild-severe anxiety. Our results are consistent with studies that have studied the same cohort population [49, 60] and studies conducted in India [32], Bangladesh [61], and the US [33, 62, 63] that have found psychosocial distress, specifically anxiety, as a prevalent phenomenon among PWID. Overall, our study highlights intersecting vulnerabilities among PWID, especially among those who identify as women and live in unstable housing conditions in the prior six months.

In this study, we tested three main hypotheses. The first hypothesis focused on examining the association between WaSH insecurity and anxiety. We found that lack of basic access to water for drinking increased the risk of anxiety among PWID regardless of the city of residence. We also found that sanitation and bathing insecurity for PWID were associated with an increased risk of anxiety in the Tijuana-San Diego border region. We found that WaSH insecurities were cumulative when assessing their relationship with anxiety: with every additional WaSH service lost, the risk of anxiety increases by 20%. This suggests that losing sanitation and bathing access would increase anxiety by 40%. Our results are consistent with other studies that have explored WaSH insecurity and psychosocial distress at the household level in other populations [16, 17, 21, 23, 47, 64–66]. In rural communities of India, for instance, sanitation insecurity, specifically open defecation practices was associated with psychosocial distress [64, 67]. At the same time, lack of drinking water and having water services shut off have been found to be significantly associated with anxiety and depressive symptoms in households of Detroit, Michigan [65]. While a few studies have examined WaSH access and anxiety, no other known study has looked at the association between each additional WaSH service lost and the incremental risk to anxiety.

Beyond the household level and considering PWID, a study conducted in the Central Appalachian Kentucky region with unhoused communities who inject drugs found that unmet WaSH needs contributed to anxiety, depression, lower self-esteem, and reinforced stigmatization [27]. These studies and our own findings suggest that WaSH insecurity contribute to a person’s inability to maintain personal hygiene and a good appearance. Concurrently, without a certain level of hygiene and appearance, one’s access to these WaSH services, needed to sustain their daily life, is restricted [5, 26, 27]. A recent study found that communities living along the Tijuana River canal are mostly deportees from the United States, unhoused people, and injection drug users that often get stigmatized for their drug use and deportee status, further limiting their access to services [28]. While our study does not fully capture WaSH-related stigma, our findings do highlight how the lack of specific WaSH services are associated with anxiety among PWID. This is important because forms of psychosocial distress can decrease the quality of life for people who also have to cope daily with the cascading effects of WaSH insecurity that amplifies experiences of stigma, exclusion, and poverty.

The second hypothesis we tested was whether individual characteristics moderated the relationship between WaSH insecurity and anxiety. In our study, we did not find any evidence to suggest that the individual characteristics tested, including city of residence, moderated the relationship between WaSH services and anxiety. We found that lack of WaSH services increases the risk of anxiety, regardless of the city in which one resides. Other studies have pointed to varying WaSH access in Tijuana and San Diego. For example, two studies in Tijuana found that communities living along the Tijuana River canal, mostly unhoused and PWID, commonly engage in open defecation and use water from the canal and *lloraderos* (spring water found at the canal’s central river channel) to meet their basic needs given their limited access to WaSH services [28, 68]. Conversely, in San Diego, a study found that unhoused communities heavily rely on business establishments and non-profit organizations to meet their basic WaSH needs [30]. In our study, we also assessed the individual characteristic of physical violence and found a marginally significant difference among those participants that reported being victims of physical violence in the past six months. This is consistent with existing studies [69, 70]. For instance, Scutella and Johnson (2018) found that an experience of recent physical violence in the 6 months among Australians experiencing homelessness was associated with considerably higher levels of psychological distress [69].

Third, our study found a significant interaction between housing status and gender. Unstably housed females are more at risk of anxiety than housed female participants. Among those who are unstably housed, females had a higher risk of anxiety than males. Our study adds to the limited research of WaSH and anxiety research among PWID and who are unhoused by highlighting the added vulnerability that unhoused women who inject drugs may experience. These findings suggest that gender intersects with housing status and may be a risk factor for anxiety among PWID, which future studies should consider when addressing health equity to improve the well-being of vulnerable populations, particularly women’s health and well-being and their access to WaSH services.

In our study, we also found that among males, housed participants have a higher risk of experiencing mild-severe anxiety compared to unstably housed male participants. The assumption we made in our study was that unstably housed participants would have higher levels of anxiety due to their reduced access to healthcare, barriers in accessing basic WaSH services, and increased risk to violence/assault from law enforcement interactions or from living outdoors [51, 71]. Our study found this to be true only for women, but not for male participants. In Australia, a study that used the same metrics to measure anxiety (GAD-7 scale) among 71 formerly unhoused people, came to a similar conclusion and found that the anxiety state of formerly homeless people did not change and in some instances increased after being housed, which alludes to the need of ongoing support services even after connecting people to housing services [72]. This suggests that being placed into housing can also mean a loss sense of belonging or disconnection from community on the street, disassociation from an environment that they have become accustomed to, and is challenging to adapt to, especially for men [73–75]. This finding could also suggest that being housed has some effect in improving emotional well-being but only among those without serious substance use problems or mental illness [76, 77]. Another study in Australia found that males re-entering housing can initially experience elevated levels of distress and find it particularly more challenging to adapt to compared to females [69].

### Limitations

While this study has contributed to our understanding of WaSH insecurity and anxiety among PWID, there are some limitations. First, our data relied on self-reported generalized anxiety using the GAD-7 scale during a two-week period, which may underestimate anxiety as an outcome. For example, there may be an underreporting of anxiety as people living in unstable housing conditions, for example, may be forced to adapt to WaSH insecurity or supress daily trauma, anxiety, and depression through substance use. Additionally, associations may not have been detected due to low statistical power based on people’s own interpretation of anxiety or stigma around it and affected responses to the questions. Our study assumed that the anxiety outcome may be related to WaSH insecurity, but it could also be related to other factors. More direct measurements of WaSH-related anxiety in future studies are needed such as the Household Water InSecurity Experiences (HWISE) scale that measures household water insecurity and includes components of WaSH-related perceived stress [78]. It is also important to note that this study does not capture all the intersectional vulnerabilities experienced among PWID, including people who identify as transgender, as only one participant was included in the sample. Also, our sample was 75% men, 25% women, further research should look at gender interactions with a larger sample of women. In this study, we also did not capture specific bathing sources used (we captured the times a person showers/bathes per week), and other forms of body hygiene including access to laundry services. Both are important measures, as the lack of these services results in stigmatization and social exclusion [26, 27] which could contribute to added levels of anxiety. Lastly, WaSH measures were limited by self-reported answers and inability to test whether the services people use are safely managed and available at all times of the day. Future studies could conduct research on the quality of WaSH services and test drinking water quality of sources unhoused communities use. More studies are needed that incorporate time as an added metric to WaSH insecurity because for communities that are unhoused services may not be available 24-h of the day. Therefore, a temporal accessibility measure is needed to capture continuous access to services to better address the unmet needs of vulnerable communities and improve access to services needed overnight.

### Recommendations

This study describes WaSH insecurity experiences among PWID in the Tijuana-San Diego border region and analyses the association between WaSH insecurity and anxiety. In this study, we address a health disparity experienced by PWID: anxiety. To improve physical and mental health outcomes among PWID that may be experiencing WaSH insecurity, there is a need to expand and invest in permanent housing, particularly in Housing First programs in both the US and Mexico, as housing and WaSH security are interconnected. Housing First provides assistance for people living in unstable housing conditions and gives them access to permanent independent housing as quickly as possible without any treatment or sobriety requirement [71, 79]. The Housing First approach has been widely integrated in high-income countries such as the US, parts of Europe, Canada, and Japan, but is lacking in places like Mexico. Expanding Housing First programs could help people who have substance use problems and help reduce the cycle of homelessness and WaSH insecurity [5, 31, 80]. To ensure housing security, wrap-around services are also needed including, access to healthcare, substance use disorder treatment, mental health services, employment services, and programs that ensure food and WaSH security. At the same time, studies have shown that gaining access to housing can reduce anxiety and suicidal thoughts among people experiencing homelessness [81, 82]. Increased criminalization among PWID and people experiencing homelessness contributes to stigmatization and reduced access to much needed services through the displacement of communities. We need to disinvest in policies and programs that criminalize vulnerable communities. WaSH security goes hand in hand with housing security. Providing people with housing can also address WaSH insecurity experienced among unhoused community members.

While housing is a complex and long-term solution, governments in both the US and Mexico need to invest in WaSH infrastructure for unhoused PWID to sustain their daily activities. In Tijuana, limited public WaSH services exist. In California, non-profit organizations play a major role in filling some WaSH service gaps and harm reduction programs to serve PWID and or unhoused communities [26, 28]. Since these populations are mobile, investing in mobile WaSH services can help serve hard to reach populations. Addressing WaSH insecurity among the most vulnerable communities, including PWID that are unhoused, can help reduce a cycle of poverty and improve the physical and mental well-being of PWID.

## Conclusion

In this study, we found that WaSH insecurity for PWID in the Tijuana-San Diego border is associated with anxiety, a form of psychosocial distress. We found that for every additional WaSH service a person lacks, the risk of anxiety increases. Lack of access to drinking water, sanitation, and shower services, were especially important predictors of anxiety among PWID in Tijuana-San Diego region. The study also found that among PWID living in unstable housing conditions, women were more likely to experience anxiety compared to men. Housing access with WaSH facilities or safe 24-h WaSH access could help reduce anxiety and health risks associated with WaSH insecurity for this population. Access to basic WaSH services for PWID who are unhoused in the US-Mexico border region are needed to improve the physical and mental health of vulnerable populations. Subsequently, the environmental health of the surrounding community may also improve.

## Data Availability

The datasets used and/or analysed during the current study are available from Dr. Steffanie A. Strathdee on reasonable request.
